# Predicting Treatment Response to Transcatheter Arterial Chemoembolization in Hepatocellular Carcinoma Patients using a Deep Learning-based Approach

**DOI:** 10.2174/0115734056367143250610045305

**Published:** 2025-06-20

**Authors:** Zhi-Wei Li, Chun-Wang Yuan, Jian Wei, Da-Wei Yang, Hui Xu, Ying Chen, Wei Ma, Zhen-Chang Wang, Zheng-Han Yang, A-Hong Ren

**Affiliations:** 1 Department of Radiology, Beijing Friendship Hospital, Capital Medical University, 95 YongAn Road, Xicheng District, Beijing, 100050, P.R. China; 2 Department of Radiology, Beijing Jianjia Rehabilitation Hospital, Building 3, Yard 6, Kangxin Road, Fengtai District, Beijing, 100071, P.R. China; 3 Center of Interventional Oncology and Liver Diseases, Beijing Youan Hospital, Capital Medical University; No.8 Xitoutiao, Youwai St, Fengtai District, Beijing, 100069, P.R. China; 4 Department of Interventional Radiography, Beijing Friendship Hospital, Capital Medical University, 95 YongAn Road, Xicheng District, Beijing, 100050, P.R. China; 5 Faculty of Information Technology, Beijing University of Technology, Beijing, 100124, P.R. China

**Keywords:** Magnetic Resonance Imaging (MRI), Prediction, Deep learning, Modified Response Evaluation Criteria in Solid Tumors (mRECIST), Receiver Operating Characteristic (ROC), Chronic Hepatitis B Virus

## Abstract

**Objectives:**

This study aimed to assess the effectiveness and precision of a deep learning-based model in forecasting the early response of HCC patients to TACE.

**Methods:**

A comprehensive review of HCC-TACE data involving 111 patients with HCC was carried out, encompassing both pre-TACE MR images (captured before the first TACE) and post-TACE imaging (acquired between 30 and 60 days following TACE). Based on the mRECIST criteria, patients were divided into two cohorts: a training dataset (91 subjects, 645 images) and a test dataset (20 subjects, 155 images). A deep learning-based model utilizing LeNet architecture with an attention mechanism was developed, targeting the prediction of HCC patients' response to TACE. The robustness and accuracy of the model were examined via ROC curves and confusion matrices.

**Results:**

Post-TACE treatment, 56 patients (50.5%) manifested an objective response (CR+PR), whereas 55 patients (49.5%) exhibited no response (SD+PD). Concerning the model's predictive ability for TACE response, the AUC was found to be 0.760 in the training dataset and 0.729 in the test dataset. The model's prediction accuracy was further corroborated by the confusion matrix, revealing an average accuracy of 70.7% in the training dataset and 72.3% in the test dataset.

**Conclusion:**

Implementing a deep learning-based model using MRI data is potent for forecasting HCC patients’ response to TACE treatment. The novel LeNet model with the attention mechanism conceived in this study contributes valuable insights that can guide the formulation of effective treatment strategies.

## 1 INTRODUCTION

Hepatocellular Carcinoma (HCC) is the fifth most common malignant tumor worldwide and represents the third leading cause of cancer-related mortality, according to estimates by the World Health Organization [1]. Chronic Hepatitis B Virus (HBV) infection, particularly prevalent in China, ranks as a prominent risk factor for HCC development [2]. Unfortunately, the disease often progresses unnoticed, with most HCC patients being diagnosed in advanced stages, bypassing the optimal treatment window due to the subtleness of early symptoms [3, 4]. This late detection leads to over 80% of the HCC patients being ineligible for surgical interventions, such as resection or liver transplantation, complicating treatment decision-making. The median survival time for patients with intermediate-stage HCC is roughly 16 months, and late-stage HCC patients usually survive a mere 6-8 months, further complicating the selection of appropriate treatment modalities [5].

Treatment strategies, including systemic therapy with sorafenib, local radiofrequency ablation, and embolization, are accessible for patients with unresectable HCC [6, 7]. Specifically, for patients with HCC in the Barcelona Clinic Liver Cancer (BCLC) stage B (intermediate stage), Transcatheter Art Chemoembolization (TACE) is widely recognized as a standard therapeutic approach [8]. However, the BCLC staging system presents significant limitations, leading to inconsistencies in therapeutic efficacy even within the same BCLC stage [9]. Consequently, nearly 60% of TACE-treated HCC patients do not benefit from this therapy, underscoring the need for alternative treatment options [10]. Thus, the ability to preoperatively predict a patient's response to TACE could substantially enhance the prognosis for HCC patients and guide the application of alternative therapies [11].

HCC patients with tumors located in segments I and IV often face difficultly achieving a complete response, therefore having a worse prognosis, while those with tumors smaller than 5 cm might have a higher likelihood of complete response [12]. Several factors can be used as predictors for early response to TACE, such as abnormal levels of AFP, arterial peritumoral enhancement, and irregular tumor margins for different sizes of tumors (between 2-5 cm or larger than 5 cm) [13]. The number of tumors can also predict the overall response to TACE [14]. Functional Magnetic Resonance Imaging (fMRI) techniques, including diffusion-weighted imaging, have shown promise in predicting early treatment response to TACE with satisfactory accuracy [15]. It has been reported that fMRI can also accurately predict early responses in HCC patients—in particular, the use of the diffusion coefficient ratio, which is calculated by the examination results of diffusion-weighted imaging performed one month after TACE—and can be used to predict progression-free survival independently [15]. Furthermore, intravoxel incoherent motion and diffusion kurtosis imaging, both of which are other emerging fMRI techniques, can also be used to predict early treatment response to TACE [16, 17]. Besides these methods, specific inflammatory biomarkers are also helpful in predicting the postoperative prognosis of HCC. Additionally, the Platelet-to-Lymphocyte Ratio (PLR) and Neutrophil-to-Lymphocyte Ratio (NLR) are also used for the prediction, especially in patients receiving the drug-eluting bead TACE (DEB-TACE) [18]. Moreover, the reliable predictors of the outcome of HCC patients receiving TACE involve a pre-treatment platelet count [19].

Radiomics, which involves extracting quantitative features from medical images, has gained attention for predicting the treatment response of HCC patients to TACE. The investigation of radiomics was performed because the accuracy of traditional statistical and machine learning methods in predicting the treatment response to TACE in unresectable HCC patients is limited [20, 21].

The deep learning technique, specifically utilizing Convolutional Neural Networks (CNNs), has emerged as an auspicious method for image classification [22]. Within the context of HCC research, deep learning-based models that employ radiological images for training have been devised. These models can not only detect tumors but also determine their staging, as well as predict prognosis [23-25]. In parallel, deep learning models using Computed Tomography (CT) images have been evaluated for predicting the effectiveness of TACE in HCC [21, 26, 27]. Nevertheless, the exploration of Magnetic Resonance Imaging (MRI)-based deep learning models remains scarce, and the precision of such models remains undefined.

In this study, an MRI-based deep learning model was formulated to address this gap, drawing data from two independent centers. This innovative model is designed to predict the treatment response of HCC patients before receiving TACE, providing predictions in a precise and noninvasive manner.

## 2 MATERIALS AND METHODS

### 2.1 Patients' Information

**Fig. (1):**
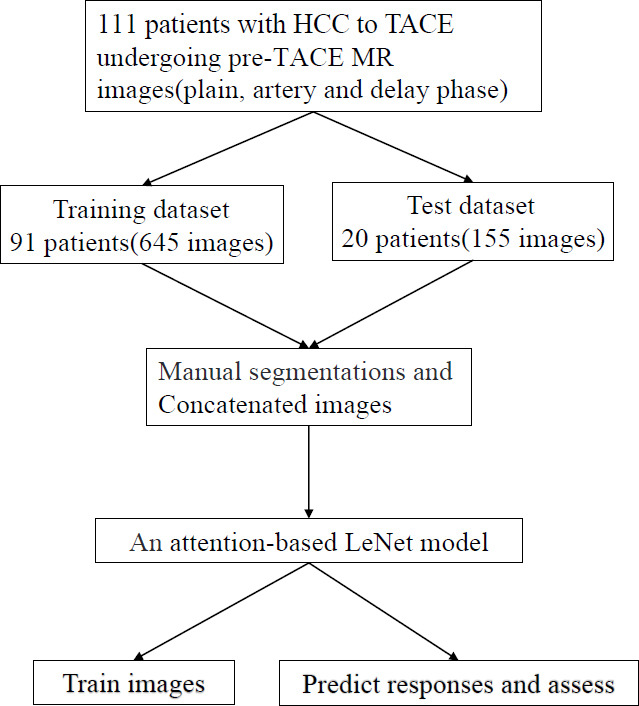
Flow diagram of the study.

Due to the retrospective nature of this study, there was no need to obtain informed consent from the enrolled subjects, but approval from the institutional review board of our hospital was obtained (2020-P2-220-01, date: October 29, 2020). All the enrolled HCC patients received TACE (first-line therapy) at two institutions, including BF Hospital (n=91) and BY Hospital (n=20), between January 2013 and December 2021. The inclusion criteria consisted of the following conditions (Fig. **S1**), also available online): (1) patients who previously received TACE treatment exclusively, without a history of any other therapy and (2) patients diagnosed with HCC based on the following criteria: the American Association for the Study of Liver Diseases guidelines, the noninvasive criteria of the Liver Imaging Reporting and Data System (LI-RADS) v. 2018, and pathological assessment. Patients were excluded if they met the following criteria: those with a history of surgical resection or systemic chemotherapy for HCC, HCC diameter of less than 1 cm with extrahepatic metastasis or portal vein invasion, bad quality of imaging, esp. in the liver region; without pre-therapy contrast-enhanced MRI within 30 days before TACE; no follow-up MRI or CT including unenhanced imaging 30-60 days after TACE; the involved lesions more than five; and diffuse or infiltrative HCCs. A total of 295 patients were initially identified. However, 184 patients were excluded through these criteria. Finally, the remaining 111 patients were enrolled in the study for further analysis (Fig. **1**).

### 2.2 Baseline Clinical Data

The clinical information for the enrolled patients was meticulously compiled from the institutional database, encompassing preoperative clinical statistics and laboratory test results. This included parameters, such as the neutrophil-to-lymphocyte ratio, absolute lymphocyte count, absolute neutrophil count, platelet-to-lymphocyte ratio, serum Alpha-Fetoprotein (AFP) level, and Platelet Count (PLT), all reflecting pretreatment values. Data were collected on the patients’ gender, age, HCC diagnosis, BCLC stage, pretreatment NLR, Child-Pugh class, and underlying causes of chronic liver disease. The continuous variables within the clinical characteristics were subsequently transformed into categorical variables, utilizing predefined cut-off values to facilitate the analysis.

### 2.3 MRI Data Acquisition before TACE

One month prior to receiving TACE, the enrolled patients received an MRI examination using a 1.5-T MRI scanner (Signa HDxt 1.5T), a product of GE Company or a 3.0-T MRI scanner, which is a product of three companies (Discovery 750w, GE; MAGNETOM Prisma, Siemens; Ingenia, Philips). All the instruments were equipped with an 8/16-element phased array coil. The liver MRI sequence details are the same with our previous article [11]. To enhance the MRI performance, an intravenous injection (i. v.) with 0.1 mmol/kg (rate, 2 mL/s) of Gadobenate dimeglumine (Magnevist, Bayer Schering Pharma AG) was administered, followed by a normal saline flush. At the following time points after contrast agent administration, dynamic T1-weighted imaging (T1WI) sequences were collected on time, including late arterial phase (25-30 seconds), portal venous phase (60-70 seconds), equilibrium phase (3-4 minutes), and delayed phase (6-8 minutes).

### 2.4 TACE Procedure

The procedure of TACE was operated by two senior interventional radiologists with clinical experience of >15 years. Based on the Seldinger technique, the target regions were examined in the direction of a Digital Subtraction Angiography (DSA) device (GE Innova 3100). Briefly, a sheath introducer was placed in the right common femoral artery. A five French (5F) angiographic catheter was inserted into the common hepatic artery in advance, and a 2.2-2.4 F microcatheter (Asahi Intecc Co. Ltd., Japan) was also inserted into the feeding hepatic artery, followed by injections of oxaliplatin (50 mg), hydroxycamptothecin (20 mg), epirubicin (50 mg), and recombinant human Interleukin-2 (2 million IU), with additional administration of lipiodol emulsion (6 ml; Lipiodol Ultra-Fluide and Guerbet, France)—the volume of which was dependent upon the tumor size and achievement of stagnant flow in the tumor-feeding artery—350-560-μm gelatin sponge particles (Hangzhou Alicon Pharmaceutical Technology Co.Ltd., Hangzhou, China), and/or a mixture of epirubicin (10 mg).

### 2.5 Acquisition of CT or MRI Data after TACE

During a period of one to two months after TACE treatment, some patients underwent CT scan examination through a multi-detector scanner device, which is a product made by several companies, including Somatom Definition AS+ 128, Siemens Healthineers (Erlangen, Germany); Aquilion one 320, Toshiba Medical Systems, (Tokyo, Japan); or Brilliance 128, Philips Healthcare (Best, Netherlands). Before contrast-enhanced scanning, patients were intravenously injected with a non-ionic contrast agent (1.5 mL/kg, Ultravist 350), which is a product of Bayer Healthcare (Berlin, Germany), and a saline flush (30-mL, and 3 mL/s of rate), sequentially. Several scanning images were captured, including those in the arterial phase (starting time was 6 seconds after reaching a trigger threshold of 100 HU at the abdominal aorta), venous phase (35 seconds after arterial phase), and delayed phase (120 seconds after venous phase). The scanning parameters were 120 kVp, 200–250 mAs, and 0.6–0.625 mm detector collimation. CT images were reconstructed in a manner of thickness/reconstruction interval of 3 mm. Some patients also underwent MR examinations 1-2 months after the TACE treatment.

### 2.6 Evaluation of Response to TACE

Between 1-2 months after the first TACE therapy, the patient response to TACE was evaluated through modified Response Evaluation Criteria in Solid Tumors (mRECIST), which was a radiological evaluation of the local lesions [28]. These patients had previously undergone multiphase MRI or CT scans. Patients were classified into four subgroups: (1) Complete Response (CR), (2) Partial Response (PR), (3) Stable Disease (SD), and (4) Local Progressive Disease (PD). Objective response was defined as CR + PR, and non-response was defined as SD + PD. Occasionally, a digital subtraction technique was used to minimize lipiodol-deposition effects. To conclude the radiological findings, three radiologists made a consensus agreement towards dispute cases.

### 2.7 Manual Segmentation of Regions of Interest

The Picture Archiving and Communication System (PACS) of BF Hospital and BY Hospital was a resource for the Contrast-Enhanced MR (CE-MR) images used in this study. As mentioned above, two senior radiologists identified the tumor Regions of Interest (ROIs) for further analysis. The ITK-SNAP software (version 3.8.0), a new way to apply advanced segmentation algorithms, was used to analyze medical images (in DICOM and NITFI formats). Those formats contained annotation along the three axes. The tumor area was determined by pre-contrast, hepatic arterial, and delayed-phase MR images. Segmentation of the three-dimensional (3D) ROIs was completed by radiologists who were blind to the therapy outcome, and ITK-SNAP—a free, open-source, multi-platform software—was used. ROIs were updated after the annotation of each layer, showing labeled three-dimensional images in the bottom left window.

### 2.8 Data Preprocessing

MATLAB 2014b (https://ww2.mathworks.cn/) was used to standardize and denoise the segmented 2D ROI image. The MR images were preprocessed using the min-max normalization (Equation **1**) to map the values between 0 and 1. Two classification labels (treatment effective and ineffective) were defined. The effective treatment included CR and PR, while SD and PD were considered ineffective. The tumor regions were manually delineated in the multiphase MR images containing lesions. The pre-contrast, hepatic arterial, and delayed-phase images were concatenated, resulting in a final image dimension of 84×84×3. A total of 645 images of 91 patients from BF Hospital were used as the training set, and 155 images of 20 patients from BY Hospital were used as the test set (1).

**Table:** 

x^*^ = *x*-*min*/*max*-*min*	(1)

### 2.9 Deep Learning Model

**Fig. (2a, b):**
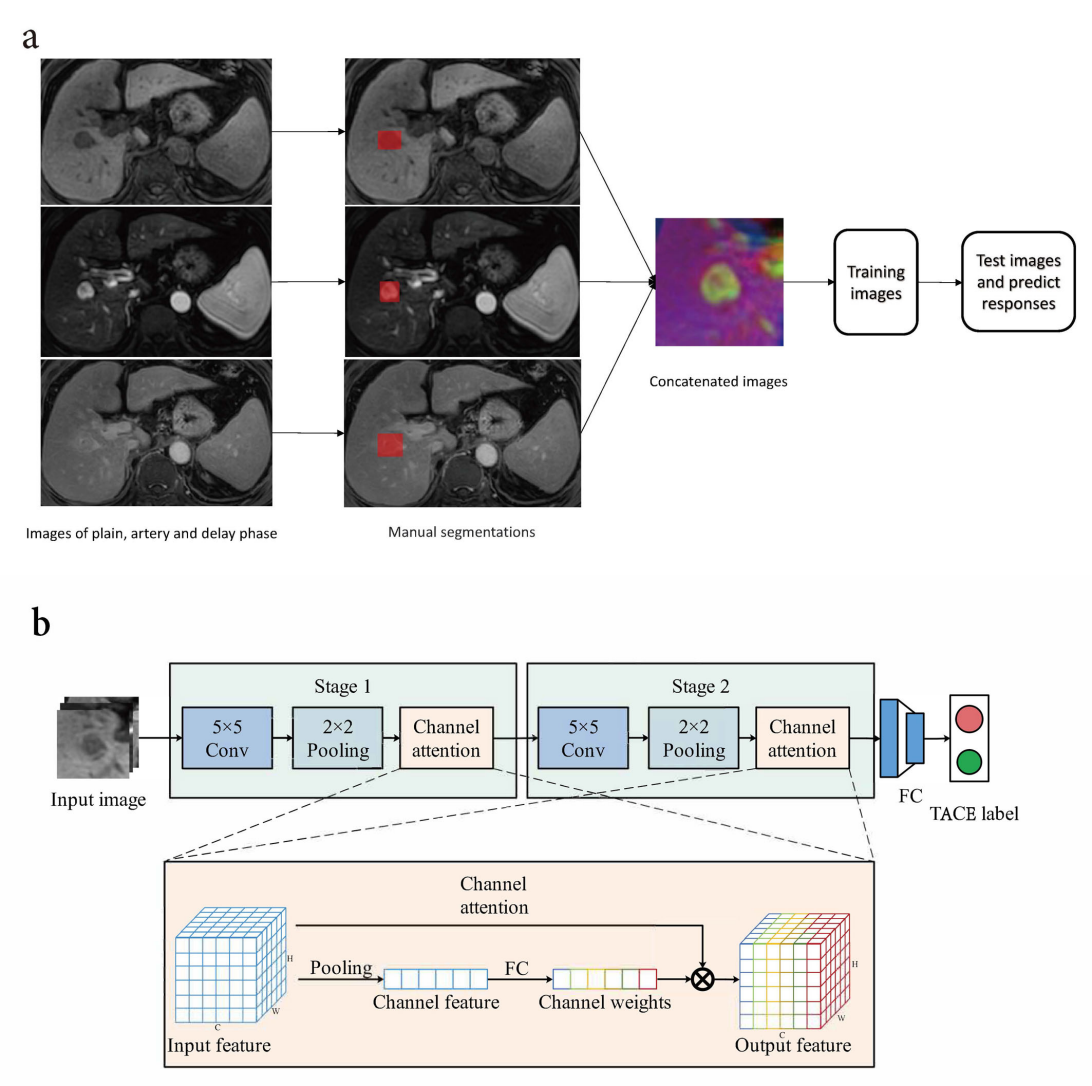
Deep learning model flowchart and the architecture of based attention LeNet. (i) 2D ROIs of HCC were manually segmented by radiologists using ITK-SNAP. We concatenated the plain phase, artery phase, and delay phase images together to form the final input data with a size of 84×84×3. Finally, 645 images of the training dataset and 155 images of the test dataset were selected to build a deep-learning model. (ii) The deep learning framework included two stages. Every stage comprises one convolution, pooling, and channel of attention. The final output layer consists of three fully connected layers, which were used to predict the response to TACE therapy.

An attention-based deep-learning model using LeNet architecture, a convolutional neural network (Fig. 2a-b), was used to predict the TACE response. This model consists of nine layers: (1) Convolutional layer C1 with six 5×5 filters, input dimension of 32×32×3, and output dimension of 28×28×6. The purpose of the convolutional layer is to extract rich features for subsequent predictions automatically. (2) Pooling layer S2, with an input size of 28×28×6 from C1 and a 2×2 and two stride filter, resulting in an output dimension of 14×14×6. The pooling layer was used to minimize feature dimensions to decrease computational complexity. (3) Attention layer A3, which employs a channel attention mechanism with the same input and output dimensions. Its purpose is to calculate the weights of different channels, allowing the model to give more attention to important channels. (4) Convolutional layer C4 with 16 5×5 filters, input dimension of 14×14×3, and output dimension of 10×10×16. (5) Pooling layer S5 with an input size of 10×10×16, and a filter 2×2, stride of 2, and output dimension of 5×5×16. (6) Attention layer A6. Similar to A3, this layer is a channel attention mechanism with the same input and output dimensions, aiming to calculate the weights of different channels while prioritizing more important ones. (7) Fully connected layer F7, having an input dimension of 400 and output dimension of 128. (8) Fully connected layer F8, having an input dimension of 128 and and output dimension of 64. (9) Fully connected layer F9, having an input dimension of 64 and an output dimension of 2, which provides the TACE efficacy prediction results. This model utilizes the Adam optimization function with a learning rate of 0.0001 and a batch size of 16. The training and prediction processes were implemented using Python with the software platform Ubuntu-18.04.1 The model of the CPU used was Intel(R) Core (TM) i7-8700K CPU @ 3.70GHz, and the GPU model was NVIDIA GeForce RTX 2080 Ti.

### 2.10 Statistical Analysis

The amassed data underwent rigorous analysis utilizing SPSS software version 27 (IBM Corporation, Armonk, NY) and GraphPad Prism 9.0. Both Chi-square tests and two-sample t-tests were employed to ascertain differences in clinical variables between the training and test datasets. To gauge the performance of the predictive model across the training and test datasets, a Receiver Operating Characteristic (ROC) analysis was conducted. The area under the ROC curve (AUC) was computed with a 95% Confidence Interval (CI) for all datasets. Furthermore, confusion matrices were formulated using Python to determine the accuracies of the TACE therapy response predictions in both the training and test datasets. Statistical significance was established at a two-tailed *P*-value of less than 0.05.

## 3 RESULTS

### 3.1 Clinical Characteristics of Patients

The study comprised a training dataset from BF Hospital, including 91 HCC patients, and an independent test dataset from BY Hospital, consisting of 20 patients. Table **1** delineates the baseline clinical characteristics of both datasets. Within the training dataset, males constituted 72 (79.1%) of the patients, while 13 (65%) males were in the test dataset. The age distribution in the training dataset spanned from 31 to 86 years (mean 61.19 ± 11.57 years), and in the test dataset, it ranged between 46 to 85 years (mean 60.60 ± 11.45 years). As for the treatment response, the training dataset was composed of 44 (48.4%) patients with CR or PR and 47 (51.6%) with SD or PD. Likewise, the test dataset featured 12 (60%) patients with CR + PR and 8 (40%) with SD + PD. An analysis of the clinical database revealed no significant differences between the two datasets.

### 3.2 Deep Learning Model Received Training and Testing for Two-Class Classification

**Fig. (3):**
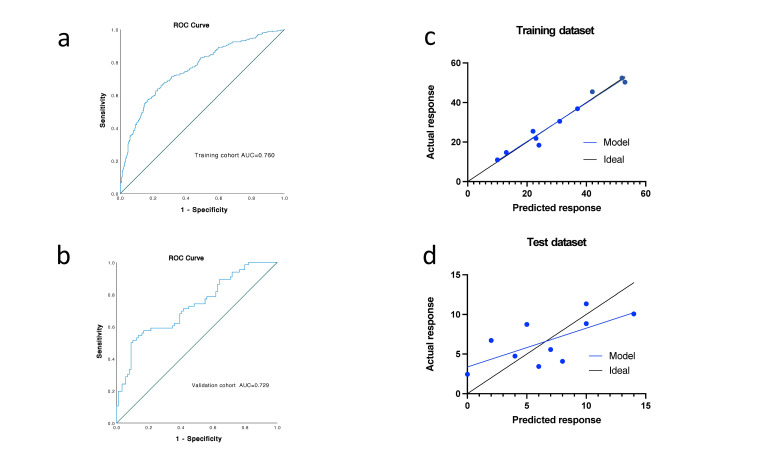
ROC curve and calibration curve for predicting the response to TACE therapy. **a**). The ROC curve of the training cohort. **b**). The ROC curve of the validation cohort. **c**). The calibration curve of the relationship between predicted and actual treatment responses in the training dataset. **d**). The correlation of actual- and predicted responses in the test dataset.

The model's fully connected layers produced multiple 2D arrays as output. When applied to the images of the training dataset (n=645), this model had an AUC of 0.760 (0.723–0.796) in predicting TACE treatment response (Fig. **3a**). Subsequently, the model was tested on the images from the independent test dataset (n=155), showing 0.729 as the value of AUC (0.649–0.810) to evaluate patient response to TACE (Fig. **3b**). These findings demonstrated that the deep learning model effectively discriminates treatment response to TACE, based on the ROI images in both datasets. The calibration curves of the data collected from the training and test datasets show a close match between the predicted and actual treatment responses (Fig. **3c**, **d**).

### 3.3 Classifiable Accuracy of Evaluation via Deep Learning Model

**Fig. (4):**
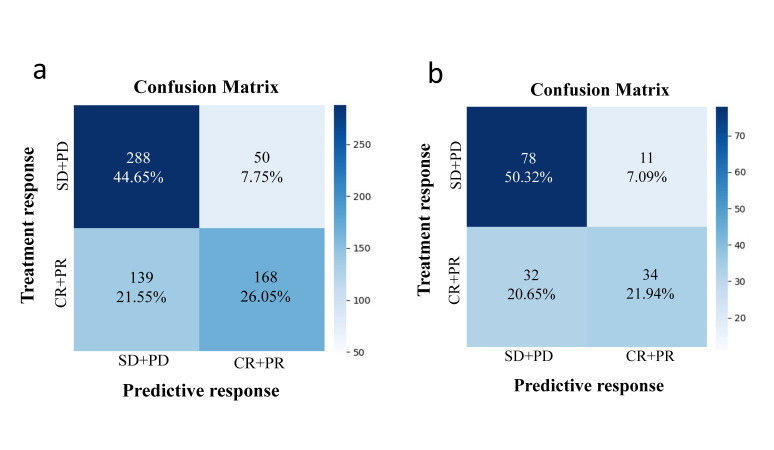
Confusion matrix for predicting the response to TACE therapy. **a**). The training dataset. **b**). The test dataset.

**Fig. (5):**
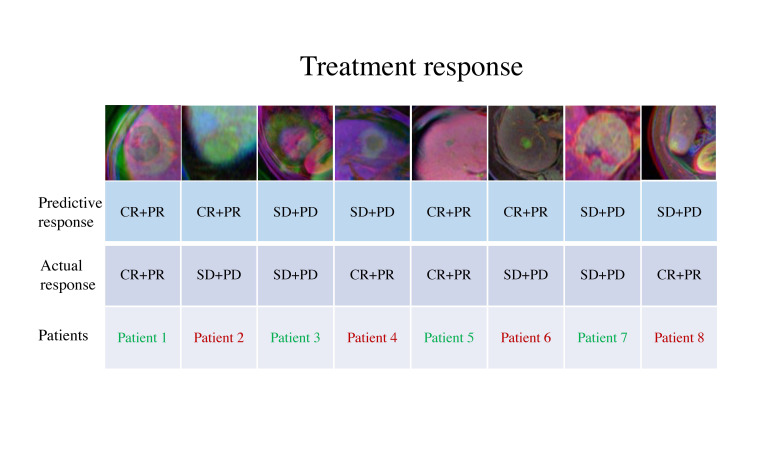
Representative images which were correctly predicted and misclassified by the LeNet model with an attention mechanism. Images belonging to patients 1-4 were in the training dataset, and patients 5-8 were in the test dataset. Green and red represent the correctly and incorrectly predicted images, respectively.

The method of confusion matrix was used to evaluate the predictive accuracy of the deep learning model to analyze each image. In predicting the objective response to TACE therapy, the average accuracy of the training dataset was 70.7% (Fig. **4a**). Similarly, the average accuracy of the independent test dataset was 72.3% (Fig. **4b**). The performances of this deep learning model in predicting therapy response were shown in Table **2**, including AUC, 95% CI, specificity, sensitivity, and accuracy between the training and test datasets. Furthermore, four representative images were selected from the training and test datasets, including correctly and incorrectly predicted images of different therapy responses (Fig. **5**). Overall, the performance of the deep learning model in predicting objective response to TACE treatment was satisfactory. However, some challenging cases were misclassified by the model. For instance, in the test dataset, patient six was incorrectly predicted as CR+PR, as well as patient eight was incorrectly predicted as SD+PD.

## 4 DISCUSSION

This research introduces a pioneering deep learning-based model to predict patient response to TACE for HCC, leveraging data from two distinct hospitals. An attention-based deep learning model combined with LeNet architecture was employed, utilizing MRI data to anticipate the effectiveness of liver cancer intervention therapy. This innovative algorithm offers a promising pathway toward enhancing precision medicine for tumor treatment through deep learning techniques. Specifically, this model, employing pretreatment ROI images of HCC patients, delivers a practical and robust approach for forecasting HCC patients' response to TACE.

Over the past five years, various retrospective studies have integrated radiomics and machine learning algorithms to predict the response of HCC patients to TACE [20, 29]. Kong *et al.* delineated a radiomics method that employed pre-TACE T2-weighted images to forecast tumor response in advanced-stage HCC patients (n=99), extracting 396 radiomics features for feature selection and model construction using a Least Absolute Shrinkage and Selection Operator (LASSO) regression model [30]. By selecting six specific radiomics features through LASSO, they accomplished a commendable prediction of TACE efficacy in advanced HCC based on radiomics and clinical data. However, this approach had several limitations, as many predefined radiomics features were manually designed, and varying scanning parameters across imaging devices could hinder reproducibility and generate non-redundant radiomics features [21].

Radiomics is a burgeoning field that extracts many features from medical images for quantitative analysis. Its convergence with machine learning algorithms fosters advancements in personalized medicine. In recent times, deep learning algorithms have demonstrated immense potential in image segmentation, recognition, classification, and reconstruction, thereby markedly boosting the robustness and accuracy of image analyses [31]. A prior study involving 789 HCC patients underscored the efficacy of a deep learning-based model, employing the Residual Convolutional Neural Network (ResNet50) in predicting patient responses to TACE treatment, with accuracies of 85.1% and 82.8% across two validation sets [21].

In contrast to current literature, this study utilizes MRI images and an attention-based LeNet model to forecast TACE efficacy. Noteworthy is the innovative integration of plain, arterial, and delayed-phase images, transcending the often-used single-phase or arterial-phase enhanced images. This comprehensive approach furnishes more exhaustive information, thereby significantly augmenting the precision of TACE response predictions.

Currently, Artificial Intelligence (AI)-based methods for predicting TACE response are categorized into two types: traditional machine learning algorithms [20, 32] and deep learning algorithms [21, 33]. The traditional machine learning algorithms for predicting TACE response first extract manually designed features, then reduce the feature’s dimensionality, and finally input the reduced features into machine learning classifiers for classification. However, the accuracy of TACE response prediction via traditional machine learning methods is usually relatively low because of the limited expressive power of manually designed features and issues, such as poor classification performance of machine learning classifiers. The second type is deep learning-based TACE response prediction methods using convolutional neural networks, which can extract features from the tumor region of patients' images and input the extracted features into fully connected layers for TACE response prediction. However, the current deep learning-based TACE response prediction methods often employ networks designed for natural image understanding, which may not fully capture the features relevant to TACE response prediction. To this end, the attention mechanisms can be used to enhance the feature representation capability of deep learning-based models for predicting TACE response. Therefore, this study developed a deep learning-based model with a channel attention mechanism commonly used in natural image understanding, improving its feature representation capability and enhancing the TACE response's prediction effectiveness.

Random forest [29] is a traditional machine-learning method that can handle high-dimensional data without requiring feature selection. This model can be rapidly trained through parallel processing because of the random selection of the feature subset, simplicity of the random forest algorithm, and independence of the trees within the Random forest. Additionally, taking the average results of all trees ensures strong overfitting resistance. However, random forest models may be composed of similar decision trees, which can obscure the actual results, and a tree may miss key features, leading to incorrect classification results and higher error rates. It is worth mentioning that Logistic regression [20] is also a classic machine-learning algorithm widely used in binary classification. This model has good interpretability, as the weights of features reflect their influence on the final results. It also has the advantages of low computational complexity and fast speed. However, logistic regression considers the distance from all data points to the decision boundary in the classification task, which can result in underfitting when dealing with unevenly distributed data. Moreover, logistic regression performs poorly when faced with many features, which may introduce significant errors in the final results. As we know, traditional machine learning requires the prior extraction of target features and relies on shallow models, which are effective for linear or simple non-linear problems. In contrast, deep learning models, due to their depth, excel in solving complex non-linear problems. This study used a LeNet-based model [34], which does not require manual feature design and only needs a prepared dataset for training. This model can extract more features with sufficient depth by operations of multiple convolutional and pooling sequences. Nevertheless, the extracted features, such as increased parameter count and overfitting, may negatively affect the network. Therefore, this study introduced SENet (a channel attention mechanism) [35] based on LeNet because SENet can differentiate between channels, with the focus of the network on the most influential features for final results while ignoring unimportant or less influential features, optimizing and simplifying the network. Consequently, the features are then passed to fully connected layers for fusion. Through backpropagation, the parameters of each layer are continuously optimized, leading to improved model performance. Finally, non-linear transformation is applied for classification, resulting in higher accuracy of deep learning compared to traditional machine learning.

Despite the innovative contributions of this study, there are several limitations to acknowledge. First, the relatively small size of the HCC patient cohort and the retrospective nature of the study—relying solely on MR images stored in the training database of a single hospital—may limit the generalizability of the findings. Future research should focus on assembling larger prospective databases, ideally encompassing multiple medical centers, to enhance the robustness and applicability of the model. Second, this study's scope was confined to training and validating the proposed model using two-dimensional MR images from only two medical centers. The restricted sample size precluded the exploration of three-dimensional MR images, which are anticipated to furnish higher accuracy and improved model quality. Consequently, including three-dimensional MR images in future research is critical to unlock more nuanced insights. Third, the correlation between the biological behavior of HCC and the predictions rendered by the deep learning network remains ambiguous and warrants further investigation. Understanding the underlying associations could refine the prediction algorithms and deepen our understanding of the disease process. Fourth, the manual delineation of the ROI constitutes a significant constraint, as the process is labor-intensive and subject to inter-observer variability. Different radiologists may identify lesions with divergent variations, which could influence disease classification. Therefore, developing an automatic segmentation model is an urgent priority for future studies. Such an innovation would minimize differences in manually annotated images of HCC patients, promoting a more standardized and efficient analytical process.

## CONCLUSION

The deep learning-based model employing MR images introduced in this study represents a pioneering approach to predicting HCC patients' response to TACE treatment. Utilizing an attention-based LeNet model enabled accurate forecasting of the early response to initial TACE treatment in HCC patients. The potential clinical application of this model extends beyond mere prediction, offering opportunities to enhance individualized clinical decision-making and inform the design of innovative treatment strategies. Future studies will continue to optimize the deep learning model to improve its accuracy of disease prediction, thereby better serving clinical practice.

## Figures and Tables

**Table 1:** Participants’ characteristics in the training and test sets.

**Characteristic**	**Training cohort** **(n=91)**	**Test cohort** **(n=20)**	***P*-value**
Patient sex	-	-	0.242
Male	72 (79.1)	13 (65)	-
Female	19 (20.9)	7 (35)	-
Age (y)	-	-	0.837
Mean, mean ± SD	61.19±11.57	60.60±11.45	-
Range	31-86	46-85	-
Causes of chronic liver disease	-	-	0.096
Hepatitis B	73 (80.2)	20 (100)	-
Hepatitis C	4 (4.4)	0 (0)	-
Alcoholic cirrhosis	6 (6.6)	0 (0)	-
Nonalcoholic fatty liver disease	3 (3.3)	0 (0)	-
Others	5 (5.5)	0 (0)	-
Child-Pugh score	-	-	0.871
A	62 (68.1)	14 (70)	-
B	29 (31.9)	6 (30)	-
BCLC stage	-	-	0.151
0+A	43 (47.3)	13 (65)	-
B+C	48 (52.7)	7 (35)	-
AFP level (ng/mL)	-	-	0.754
< 20	58 (63.7)	12 (60)	-
≥ 20	33 (36.3)	8 (40)	-
Diameter of largest nodule	-	-	0.779
< 5 cm	69 (75.8)	16 (80)	-
≥ 5 cm	22 (24.2)	4 (20)	-
HCCs per patient	-	-	0.824
1	43 (47.3)	10 (50)	-
≥ 2	48 (52.7)	10 (50)	-
Pretreatment ANC (10^9^/L), mean ± SD	2.85±1.25	2.77±1.74	0.807
Pretreatment ALC (10^9^/L), mean ± SD	1.43±0.77	1.74±0.81	0.109
Pretreatment NLR < 5 ≥ 5	85 (93.4) 6 (6.6)	20 (100) 0 (0)	0.589
Pretreatment PLT (10^9^/L), mean ± SD	120.42±80.26	138.35±74.60	0.345
Pretreatment PLR, mean ± SD	94.22±70.46	78.50±22.71	0.327
Response to therapy	-	-	0.345
CR+PR	44(48.4)	12(60)	-
SD*+PD	47(51.6)	8(40)	-

BCLC stage, Barcelona Clinic Liver Cancer stage; AFP, alpha-fetoprotein; ANC, Absolute neutrophil count; ALC, Absolute lymphocyte count; NLR, Neutrophil-to-lymphocyte ratio; PLT, Platelet; PLR, Platelet-to-lymphocyte ratio; SD, Standard deviation; CR, Complete response; PD, Progressive disease; PR, Partial response; SD*, Stable disease. Unless otherwise indicated, data are the number of patients, and data in parentheses are percentages.

**Table 2:** Performance of MRI-based deep learning model in predicting treatment response.

**Groups**	**AUC**	**95%CI**	**Sensitivity (%)**	**Specificity (%)**	**Accuracy** **(%)**	***p*-value**
Training cohort	0.760	0.723~0.796	63.8	77.2	70.7	< 0.001
Validation cohort	0.729	0.649~0.810	51.5	89.9	72.3	< 0.001

**Abbreviations:** AUC, the area under the receiver operating characteristic; CI, the confidence interval.

## Data Availability

The datasets used and/or analyzed during the current study are available from the corresponding author [A-H.R] upon reasonable request.

## LIST OF ABBREVIATIONS

MRI: Magnetic Resonance Imaging
ROC: Receiver Operating Characteristic
HCC: Hepatocellular Carcinoma
HBV: Chronic Hepatitis B Virus
BCLC: Barcelona Clinic Liver Cancer
TACE: Transcatheter Arterial Chemoembolization
CT: Computed Tomography
AFP: Alpha-Fetoprotein
PLT: Platelet Count
DSA: Digital Subtraction Angiography

## STANDARDS OF REPORTING

STROBE guidelines were followed.
