# Supplementary material for: Predicting Treatment Response to Transcatheter Arterial Chemoembolization in Hepatocellular Carcinoma Patients using a Deep Learning-based Approach
Source: Curr Med Imaging. 2025 Jun 20;21:e15734056367143. doi: 10.2174/0115734056367143250610045305 (PMC13223422; doi:10.2174/0115734056367143250610045305)
Supplement: Supplementary file 1 [file CMIM-21-E15734056367143_SD1.pdf]

1573-4056/25

Send Orders for Reprints to reprints@benthamscience.net

BENTHAM SCIENCE

CrossMark

Current Medical Imaging

Content list available at: https://benthamscience.com/journals/cmir

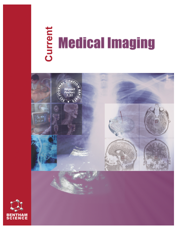

Predicting Treatment Response to Transcatheter Arterial Chemoembolization in Hepatocellular Carcinoma Patients using a Deep Learning-Based Approach

Zhi-Wei Li[1,2], Chun-Wang Yuan[3], Jian Wei[4], Da-Wei Yang[1], Hui Xu[1], Ying Chen[5], Wei Ma[5], Zhen-Chang Wang[1], Zheng-Han Yang[1] and A-Hong Ren[1,*]

[1]Department of Radiology, Beijing Friendship Hospital, Capital Medical University, 95 YongAn Road, Xicheng District, Beijing, 100050, P.R. China
[2]Department of Radiology, Beijing Jianjia Rehabilitation Hospital, Building 3, Yard 6, Kangxin Road, Fengtai District, Beijing, 100071, P.R. China
[3]Center of Interventional Oncology and Liver Diseases, Beijing Youan Hospital, Capital Medical University; No.8 Xitoutiao, Youwai St, Fengtai District, Beijing, 100069, P.R. China
[4]Department of Interventional Radiography, Beijing Friendship Hospital, Capital Medical University, 95 YongAn Road, Xicheng District, Beijing, 100050, P.R. China
[5]Faculty of Information Technology, Beijing University of Technology, Beijing, 100124, P.R. China

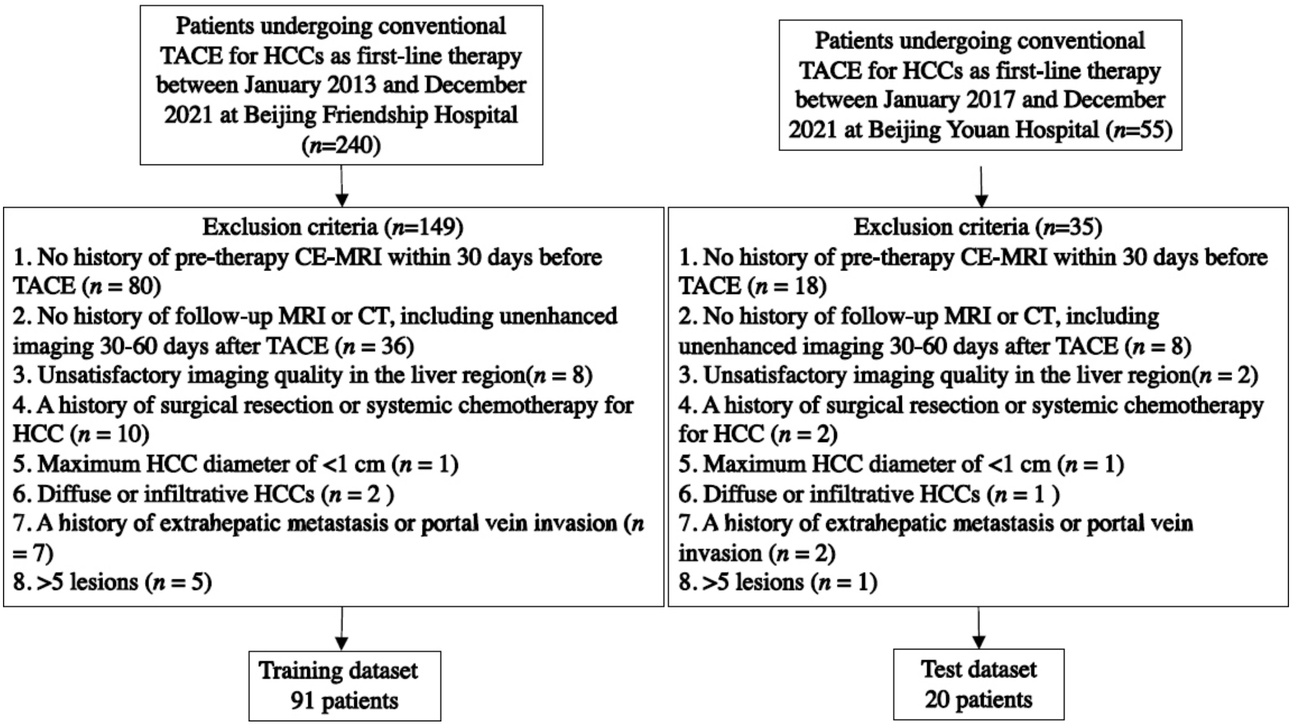

Fig. (S1). Study flow chart 1.

DOI: 10.2174/0115734056367143250610045305, 2025, *21*, e15734056367143

© 2025 The Author(s). Published by Bentham Science Publisher.

This is an open access article distributed under the terms of the Creative Commons Attribution 4.0 International Public License (CC-BY 4.0), a copy of which is available at: https://creativecommons.org/licenses/by/4.0/legalcode. This license permits unrestricted use, distribution, and reproduction in any medium, provided the original author and source are credited.
